# Supplementary material for: Magnitude, risk factors and economic impacts of diabetic emergencies in developing countries: A systematic review
Source: PLoS One. 2025 Feb 4;20(2):e0317653. doi: 10.1371/journal.pone.0317653 (PMC11793792; doi:10.1371/journal.pone.0317653)
Supplement: S1 Table — (PDF) [file pone.0317653.s001.pdf]

Supporting information(S1)

S1 Table: Qualitative analysis of included studies describing the geographic location, type of study, sample size, population and associated risk factors of diabetic emergencies

Author	year	Name of Developing countries	Study type	DMEs Sample size(n)	Population (DM type)	Risk factors		
						DKA	HHS	S.Hypoglycemia
(56)N.Lotter et al.,	2021	Captown, S.Africa R	24-week Retrospective	197	T1andT2	Infection (29.4%) Non-compliance(Medication, alcohol Diet		Non-compliances (Medication alcohol Diet)
(57) Jasper, et al.,	2014	Jos, Nigeria	Retrospective	2470	T1	Poor compliance with medications new diabetes, T1DM		Poor compliance with medications
(70)Ndizihiwe, Eulade	2021	Teritioary hospitals, Rwanda	prospective	143	T1andT2	Infect ions, newly diagnosed DM and poor drug adherence		
(108)Kakusa, M. et al	2016	Lusaka, Zambia	Cross sectional	80	T1andT2	Treatment noncompliance, new detection of diabetes, infections		
(59)Edo A E.,	2012	Legos, Nigeria	Registry	84	T1and T2	Malaria,Infection Drug non Adherence		
(60)Mbugua, P. K.,et al.,	2005	Nairobi, Kenya	Nine-month prospective study	648	T1and T2	Missed insulin(34%), overt infection (23.4%)		
(61)Negera , et al,	2020	Jimma, Ethiopia		348	T1and T2	Comorbidity, type 1 DM, uncontrolled blood glucose (AOR 1.91) and lack of access to a health facility within a reasonable distance (AOR 1.96)		

(62)Rudasingwa , GJ., et al.,	2012	Kigali, Rwanda	Cross-sectional	294	T1and T2	T1DM, younger age, illiteracy, new diabetes, and infection had significant association		
(109)Ndebele NFM .et al.,	2018	S. Africa, KwaZulu-Natal	10-month prospective	105	T1and T2	infection, poor adherence to treatment		
(71)Bassili A.,et al.,	2001	Egypt	5-Year Facility-Based Retrospective Study	134	T1	Children with lower educational levels, uninsured and living in semiurban and rural residence,		
(64)Bateganya , M. H.et al,	2003	Mulago Uganda	Cross sectional	3103	T1and T2	Omission of insulin and intercurrent infections (UTI and pneumonia)		
Jay A., et al	2004	India	Retrospective	68	T1	Mainly DKA: new-onset diabetes with sepsis (37%), new-onset diabetes alone (31%), insulin omission (15%), and infection with insulin omission (7%)		
(66)Desse T.A, et al.	2015	Jimma, Ethiopia	Retrospective	421	T1 and T2	Infections (59 %), non-compliance to medications (32.3 %), and newly diagnosed diabetes (23.6 %)		
(74) Li J. et al.,	2014	Guangzhou, China	cross-sectional	611	T1	female gender (RR)= 2.12], medical reimbursement rate<50% (RR=1.84), uncontrolled diet (RR = 1.76), (‘never controlled’ or ‘sometimes controlled’ vs .‘usually controlled’, or 2.02,respectively), , presence of neuropathy(RR =1.89), smoking (RR=1.48) and		16 male gender (RR = 1.71).2 smoking (RR = 2.18) and , medical reimbursementrate <50% (RR = 1.36) longer duration of T1DM (per 5-year increase, RR = 1.22) exercise<150 min/week (RR=1.66) lower HbA1c values (per 1.0% decrease, RR=1.46)underweight (RR = 1.44),

								uncontrolled diet (RR = 2.09)
(75)Al-Obaidi, et al.,	2019	Basrah, Iraq	cross-sectional	147	T1	Younger age, underweight, being without a job, low personal and/or mother educational level, travel, home glucose monitoring less than 7 times a week, uncontrolled HbA_{1c} and insulin stoppage.		
(68)Adem A.,	2011	Addis Ababa, Ethiopia	4 year retrospective study	724	T1 and T2	infections (36.3%). (Among those with infection, pneumonia (9.9%), UTI (8.1%), diabetic foot ulcer (9.7%), tuberculosis (5.5%)		
(69)Abate, M. D.et al.,	2023	Bahrdar, Ethiopia	5yr Multicenter retrospective	453DKA= HHS=	T1 and T2	Diabetes duration of ≥ 3 years, recent acute illness, presence of comorbidity, poor glycemic control, history of medication non-compliance, and without community health insurance were significant predictors		
(110)Rahim M.,et al.	2018	Bangladish, Dhaka	cross-sectional	200	T1and T2	Infection, Non-compliance of antidiabetic meds,<br>Acute illnesses		
(72)Ponesai, Net al.,	2015	Chirumanzu, Zimbabwe	Case control	68	T1andT2			Over Insulin therapy [OR=3.83], Missing doses [OR=6.63]and co-morbidity with hypertension

								[OR=4.10], Distance from hospital >5km [3.97, 95%CI 1.77-9.00)] and failure to get drugs
(76) Wu X-y ,et al.	2020	Shanghai, China	5year retrospective	158	T2	Infections (70.3%), new diabetes (17.7%) and non-compliance to medications (5.7%)		
