# Supplementary material for: Magnitude, risk factors and economic impacts of diabetic emergencies in developing countries: A systematic review
Source: PLoS One. 2025 Feb 4;20(2):e0317653. doi: 10.1371/journal.pone.0317653 (PMC11793792; doi:10.1371/journal.pone.0317653)
Supplement: S2 Table — A: Risk of bias assessment of the studies included in this systematic review, B: Methodological Quality of the economic evaluation studies. (ZIP) [file pone.0317653.s002.zip › S2 Table A.pdf]

S2 Table A: Risk of bias assessment of the studies included in this systematic review

Newcastle-Ottawa Quality Assessment Scale for Cohort (retrospective and prospective) Studies									
Study	Selection				Comparability	Outcome			Total score (max 8)
	Representativeness of the exposed cohort	Selection of non-exposed cohort	Ascertainment of exposure	Demonstration that outcome of interest was not present at start of study	Comparability of cohorts	Assessment of outcome	Adequacy of follow up	Length of follow up	
[56]Lotter (2021)	*	*	*			*			****
[57]Jasper (2014)	*	*	*		*	*			*****
[63]Iradukunda (2021)	*	*	*		*	*			*****
[60]Mbugua 2005	*	*	*		*	*	*	*	*******
[18]Ogbera 2007	*	*	*		*	*	*	*	*******
[76]Wu, X-y. 2020	*	*	*		*	*			*****
[66]Desse(2015)	*	*	*		*	*			*****
[20]Sarfo-Kantanka(2016	*	*	*		*	*			*****
[65]Ogbera, 2009	*	*	*		*	*	*	*	*******
[68]Adem A. 2011	*	*	*		*	*			*****
[69]Abate2023	*	*	*		*	*			*****
[19]Ajayi, E. A. 2009	*	*	*		*	*			*****
[77]Ahmed MM.2014	*	*	*		*	*	*	*	******
[70]Ndizihiwe, 2021	*	*	*		*	*	*	*	*******

Notes: The Newcastle-Ottawa Quality Assessment Scale for Cohort Studies was used. The total number of stars possible was 8. Quality assessment was done by 1 investigator (GT), and any uncertainties were discussed with other investigators (HK and MB).

Newcastle-Ottawa Quality Assessment Form for Cohort Studies

Note: A study can be given a maximum of one star for each numbered item within the Selection and Outcome categories. Amaximum of two stars can be given for Comparability.

Reviewer:______________________________________Date________________________________
_Author____________________________________Year_________ Record Number_________

Selection

1) Representativeness of the exposed cohort

- ***a)*** Truly representative ***(one star)***
- ***b)*** Somewhat representative ***(one star)***
- c) Selected group
- d) No description of the derivation of the cohort

2) Selection of the non-exposed cohort

- ***a)*** Drawn from the same community as the exposed cohort ***(one star)***
- b) Drawn from a different source
- c) No description of the derivation of the non exposed cohort

3) Ascertainment of exposure

- ***a)*** Secure record (e.g., surgical record) ***(one star)***
- ***b)*** Structured interview ***(one star)***
- c) Written self report
- d) No description
- e) Other

4) Demonstration that outcome of interest was not present at start of study

- ***a)*** Yes ***(one star)***
- b) No

Comparability

1) Comparability of cohorts on the basis of the design or analysis controlled for confounders

- ***a)*** The study controls for age, sex and marital status ***(one star)***
- ***b)*** Study controls for other factors (list)___________________________________***(one star)***
- c) Cohorts are not comparable on the basis of the design or analysis controlled for confounders

Outcome

1) Assessment of outcome

- ***a)*** Independent blind assessment ***(one star)***
- ***b)*** Record linkage ***(one star)***
- c) Self report
- d) No description
- e) Other

2) Was follow-up long enough for outcomes to occur

- ***a)*** Yes ***(one star)***
- b) No

Indicate the median duration of follow-up and a brief rationale for the assessment above:

____

3) Adequacy of follow-up of cohorts

- ***a)*** Complete follow up- all subject accounted for ***(one star)***
- ***b)*** Subjects lost to follow up unlikely to introduce bias- number lost less than or equal to 20% or description of those lostsuggested no different from those followed. ***(one star)***
- c) Follow up rate less than 80% and no description of those lost
- d) No statement

Thresholds for converting the Newcastle-Ottawa scales to AHRQ standards

(good, fair, and poor):

- **Good quality:** 3 or 4 stars in selection domain AND 1 or 2 stars in comparability domain AND 2or 3 stars in outcome/exposure domain

- **Fair quality:** 2 stars in selection domain AND 1 or 2 stars in comparability domain AND 2 or 3stars in outcome/exposure domain

- **Poor quality:** 0 or 1 star in selection domain OR 0 stars in comparability domain OR 0 or 1 starsin outcome/exposure domain

JBI Critical Appraisal Checklist used for Analytical Cross-sectional Studies

Reviewer:____________________________ Date:_________Year_________ Record Number_________

	Yes	No	Unclear	Not applicable
1. Were the criteria for inclusion in the sample clearly defined?	□	□	□	□
2. Were the study subjects and the setting described in detail?	□	□	□	□
3. Was the exposure measured in a valid and reliable way?	□	□	□	□
4. Were objective, standard criteria used for measurement of the condition?	□	□	□	□
5. Were confounding factors identified?	□	□	□	□
6. Were strategies to deal with confounding factors stated?	□	□	□	□
7. Were the outcomes measured in a valid and reliable way?	□	□	□	□
8. Was appropriate statistical analysis used?	□	□	□	□

Overall appraisal: Include □ Exclude □ Seek further info □

Comments (Including reason for exclusion)

____

____

____.
