# Supplementary material for: Magnitude, risk factors and economic impacts of diabetic emergencies in developing countries: A systematic review
Source: PLoS One. 2025 Feb 4;20(2):e0317653. doi: 10.1371/journal.pone.0317653 (PMC11793792; doi:10.1371/journal.pone.0317653)
Supplement: S2 Table — A: Risk of bias assessment of the studies included in this systematic review, B: Methodological Quality of the economic evaluation studies. (ZIP) [file pone.0317653.s002.zip › S2 Table B.pdf]

S2 Table B: Methodological Quality of the economic evaluation studies

Assessments tools methodological questions	All studies			[84]Ha WC.,et al.	[80]Mutsa P. et al	[83]Kansra, P. et al	[82]Acharya, L. D.et al.,	[79]Naser, A. Y. et al.,	[44]Feleke, Y. et al	[81]Aljunid SM., et al.	[43]Assefa, B., et al.,
	Y	P	N	(Y)(P)(N)	(Y)(P)(N)	(Y)(P)(N)	(Y)(P)(N)	(Y)(P)(N)	(Y)(P)(N)	(Y)(P)(N)	(Y)(P)(N)
1.Was a clear definition of the illness given?	8	0	0	Y	Y	Y	Y	Y	Y	Y	Y
2. Were epidemiological sources carefully described?	7	1	0	Y	Y	Y	P	Y	Y	Y	Y
3. Were costs sufficiently disaggregated?	3	4	1	Y	Y	P	P	P	Y	N	P
4. Were activity data assessed appropriately?	5	1	2	Y	Y	N	Y	Y	N	Y	N
5. Were the sources of all cost values analytically described?	4	2	2	P	Y	P	Y	N	Y	Y	N
6. Were unit costs appropriately valued?	7	1	0	Y	P	Y	Y	Y	Y	Y	Y
7. Were the methods adopted carefully explained?	4	3	1	Y	Y	P	P	N	P	Y	Y
8. Were costs discounted?	6	0	2	Y	N	Y	Y	Y	Y	N	Y
9. Were the major assumptions tested in a sensitivity analysis?	4	1	3	Y	Y	P	Y	Y	N	N	N
10. Was the presentation of study results consistent with the methodology of the study?	5	2	1	Y	Y	Y	N	Y	P	Y	P
Total score by study *	53	15	12	(9)(1)(0)	(8)(1)(1)	(5)(4)(1)	(6)(3)(1)	(7)(1)(2)	(6)(2)(2)	(7)(0)(3)	(5)(2)(3)

**** Total score by study was the sum of all answers: Partially (P), Yes(Y)(1) No(N)(0)***
