# Supplementary material for: Magnitude, risk factors and economic impacts of diabetic emergencies in developing countries: A systematic review
Source: PLoS One. 2025 Feb 4;20(2):e0317653. doi: 10.1371/journal.pone.0317653 (PMC11793792; doi:10.1371/journal.pone.0317653)
Supplement: S2 File — (PDF) [file pone.0317653.s004.pdf]

Section and Topic	Item #	Checklist item	Location where item is reported
**TITLE**			
Title	1	Magnitude, risk factors and economic impacts of diabetic emergencies in developing countries: A systematic review	Title
**ABSTRACT**			
Abstract	2	**Background:** Diabetic ketoacidosis (DKA), hyperglycemic hyperosmolar syndrome (HHS) and sever hypoglycemia are considered as the life-threatening diabetic emergencies of diabetic patients worldwide. As the prevalence of diabetes grows in developing countries, so too does the impact of these costly human and economic complications. Noticeable scarcity of data concerning the magnitude, the cost expenditures as well as well unidentified predictors of these complications made the management more difficult in the resource limited health care settings. Thus, this systematic review aimed to assess the magnitude, risk factors and economic impacts of diabetes emergencies among diabetic patients in the developing countries.<br>**Methods:** Following PRISMA (2020) guidelines, databases of PubMed, EMBASE, Cochrane and Scopus were searched for studies reporting on prevalence, risk factors, and direct costs of diabetes emergencies published in English from 2000 to 2023. Forty eligible studies were extracted and retrieved using manual data extraction form and automation tools. Studies were analyzed and combined in a narrative synthesis. The estimations of direct cost expenditure were standardized to 2023 USD.<br>**Result:** A comprehensive examination was conducted on the 40 eligible studies, with the majority originating from African sources. The review shows the prevalence of diabetic emergencies; DKA episodes in the range of (3.8%-73.4%), HHS (0.9%-58%) and Sever hypoglycemia (3.3%-64.7%) per year in the developing countries. Infection, new onset of the diabetes, and non-compliance to medications and diets were reported as the most common risk factors of theses diabetic emergencies. Besides, the costs of hospitalization taken from the patients' perspective, that were associated per one diabetic emergency event per patient was reported in the range of 105-230 USD in the developing countries.<br>**Conclusion:** we need to tailored strategies towards prevention and management to reduce the occurrence of the diabetes emergencies events as the prevalence rate has arising and the reported associated factors are common in the limited health care setting. Further investigation on the specific aspects of identified risk factors of these nations would be required. diabetic emergencies impose a high economic burden to individuals and the societies. Thus, standardize resources allocation mainly to diabetes mullites and specifically to its complications seems mandatory in the developing countries.<br>*Key words: Diabetes Mullites, Acute Complications Emergencies, Prevalence, Risk Factors, Cost*	Abstract
**INTRODUCTION**			
Rationale	3	In developing countries, where a substantial proportion of diabetics fall within the 45-65 age range, these emergencies lead to a considerable economic burden, hampering productivity and straining economies (36-38). The direct medical expenses and indirect costs associated with DKA and HHS are substantial, further exacerbated by limited allocations for diabetes treatment in national health budgets (39-47). Despite the escalating occurrence	Last paragraph of the introduction

Section and Topic	Item #	Checklist item	Location where item is reported
		of diabetic emergencies in developing nations, noticeable scarcity of studies, a lack of specificity and statistical clarity in existing studies necessitates a comprehensive review.	
Objectives	4	This manuscript aims to address this gap by synthesizing information from studies conducted since the year 2000, focusing on the prevalence, risk factors, and economic burden of diabetic emergencies among known diabetic patients. This review seeks to contribute essential insights to the understanding of challenges, prevention strategies, and management practices related to diabetic emergencies in developing countries.	Last paragraph of the introduction
**METHODS**			
Eligibility criteria	5	Studies that used observational (cross-sectional, case– control, prospective and retrospective cohorts) epidemiological designs involving known diabetic patients in the developing countries setting were considered. Articles published from 2000 to 2023 in the English language with full text and reported original research findings from the developing country based on the 2023 world bank designations, that addressed one or more of the focused areas of the review were included.	Eligibility criteria
Information sources	6	We searched PubMed, COCRAINE, MEDLINE, EMBASE, SCOPUS for studies on prevalence, risk factors, and cost of diabetes emergencies published in English from 2000 to 2023. Other sources such as Google Scholar were hand-searched for additional studies and we included 40 studies for our analysis. In addition, references of included articles were hand searched for any other original articles.	Data source and search strategies
Search strategy	7	A search strategy was developed for all databases using key concepts in the research question: Diabetes Mullites emergencies, acute complications, prevalence, Risk factors and economic burden, cost and developing countries.	
Selection process	8	Titles and abstracts were examined for inclusion by two reviewers (HK and TG). Full copies of papers which appeared to fulfil the inclusion criteria were obtained and were independently selected by the reviewers for inclusion in either phase of the review. Disagreements were resolved by discussion. Reviews, editorials, commentaries and opinion pieces (all non-peer-reviewed publications) were excluded. Lastly, papers that provided only abstracts or vague reporting of methods or results, non-rigorous sampling design, unsuitable outcome definitions or severe methodological limitations in their statistical analyses were also excluded.	Study selection
Data collection process	9	Manual data extraction form as well as Covidence and EPPI-Reviewer automation tools were utilized and pilot tested, with adaptations made accordingly. For every study, the fist reviewer (HK) separately retrieved the following information when data were available: Data for collection includes title, authors, study design, publication year and country, sample size, participant type of DM and major findings of the interested outcomes. Any disagreements regarding data extraction were resolved through discussions with a second reviewer. Then extracted studies checked for accuracy by a senior researcher (TG) for final inclusion of the studies. Disagreements were resolved through discussion and with reference to the original article.	Data extraction

Section and Topic	Item #	Checklist item	Location where item is reported
Data items	10a	This systemic review includes the studies and reports that describe the diabetic emergencies as<br>**Diabetic ketoacidosis (DKA**) was defined based on a clinical diagnosis of DKA when there is RBGL of > 250 mg/dl, urine ketone body of ≥ +2, Arterial pH of < 7.3, and Bicarbonate of < 15 meq/l.<br>**Hyperglycemic Hyperosmolar Syndrome** (HHS was considered when RBGL is > 600 mg/dl, with alteration in mental status with minimal or absent urine ketone body in diabetic patients.<br>**Sever Hypoglycemia**: described as a diabetic patient required assistance of another person and visited emergency health care settings for management.<br>**Cost:** was defined as the total direct costs (expressed in 2023 USD) expensed for the diagnosis, medical treatment, and hospitalizations associated with the diabetic emergencies (DKA, HHS and sever hypoglycemia) of the diabetic patients.	Description of the interested outcomes
Study risk of bias assessment	11	We employed specific assessment tools tailored to each study design to evaluate the quality of individual studies. For cross-sectional studies, we utilized the Joanna Briggs Institute (JBI) evidence synthesis critical appraisal checklist, as recommended by Moola S. and colleagues. Additionally, for cohort studies, the Newcastle-Ottawa Quality Assessment Scale, as advocated by Wells G., was applied. Furthermore, studies focusing on the evaluation of cost expenditures related to diabetic emergencies underwent assessment using the British Medical Journal economic evaluation checklist developed by Drummond. Any disagreements in the assessment process were resolved through discussion.	Quality assessment
**RESULTS**			
Study selection	13a&b	The primary search strategy identified 962 articles, with 392 duplicates subsequently excluded. Out of the remaining 570 articles, 105 were eliminated due to reasons for diabetic emergencies were associated with some expected factors. Additionally, 179 articles were removed during the initial title screening due to lack of information, and 161 were excluded during the second level of abstract screening because of irrelevant conte of the interested outcomes, resulting in 103 articles for full-text evaluation. Among these 125 studies, 65 were excluded for various reasons, including non-English language, conference presentations, unavailability of the full text, lack of peer review, or being a literature review on diabetes.	Result 1st paragraph
Study characteristics	14	Each included studies (40) are presented in diagram	
Certainty of evidence	15	Assessments of certainty (or confidence) in the body of evidence for each outcome were stated and assessed.	Last paragraph of the rsult
**DISCUSSION**			
Discussion	16a	Our findings suggested that, despites their magnitude were huge, DMEs prevalence were varied inter and intra of the developing countries.<br>The compiled finding of this of systematic review showed that DKA is the predominate diabetic emergencies	In each sub title of the interested

Section and Topic	Item #	Checklist item	Location where item is reported
		among younger age and T1DM diabetic patients ranging 3.8% in China to 73.4% in Ethiopia. In consistent with this review a large survey in UK, find that infection was identified as the most common precipitating factor for diabetic ketoacidosis (45%), followed by insulin omission (20%); other causes included newly diagnosed diabetes and alcohol or drug related problems.	outcomes
	16b	The exclusion of articles not written in English could have led to the omission of relevant articles in the area under study. The review specifically concentrated on peer-reviewed articles and omitted grey literature, such as academic theses.	Limitation
	16c	Studies that included in this review are identifying predictors of diabetic emergencies; however, they have been in mixed populations of patients with T1DM and T2DM. Thus, reporting separately in children and adolescents, or in other highly selected populations may limit their generalizability.	
	16d	Recognizing individuals at greater risk of diabetic emergencies is crucial for healthcare providers to implement tailored strategies, ultimately reducing emergency visits and hospital admissions. Moreover, the economic implications revealed in the review emphasize the substantial burden diabetic emergencies impose on national healthcare systems in developing countries. Consequently, integrating preventive measures into diabetes management programs is imperative, not only for the well-being of affected individuals but also to alleviate the significant strain on limited health resources and expenditures in these nations.	Conclusion
**OTHER INFORMATION**			
Registration and protocol	17a	The protocol for this review has been registered in PROSPERO (ID: CRD42023494195).	1st paragraph of the methodology
	17b	the review protocol can be accessed at https://www.crd.york.ac.uk/prospero/#searchadvanced.Halefom Kahsay.	
	17c	No amendments at registration or in the protocol	
Support	18	No financial support is provided to this review	
Competing interests	19	No conflict of interest between the authors	
Availability of data, code and other materials	20	template data collection forms; data extracted from included studies; data used for all analyses; analytic code; any other materials used in the review are found in the hands of the corresponding author (HK).	
