# Supplementary material for: Magnitude, risk factors and economic impacts of diabetic emergencies in developing countries: A systematic review
Source: PLoS One. 2025 Feb 4;20(2):e0317653. doi: 10.1371/journal.pone.0317653 (PMC11793792; doi:10.1371/journal.pone.0317653)
Supplement: Attachment — Submitted filename: 1. Response to Reviewers.docx [file pone.0317653.s005.docx]

**Response to Reviewer**

*Dear Reviewers!*

*Thank you for taking the time to review our manuscript titled* "Magnitude, risk factors, and economic impacts of diabetic emergencies in developing countries: A systematic review." *We sincerely appreciate your thoughtful feedback and constructive suggestions. We have carefully reviewed your comments and made the necessary revisions to strengthen our manuscript. Below, we provide detailed responses to each point raised.*

*Regards,*

*Halefom Kahsay*

**Response to Reviewer #1**

Reviewer #1: Thank you for submitting interesting paper.
I have the following concerns.
1. Abstract
・Some words are misspelled. For example, sever is severe, and Diabetes Mullites is Diabetes Mellitus.
**Response**: Revised and Corrected
2. Introduction, Data Sources and search strategies
「We searched PubMed, COCRANE, MEDLINE, EMBASE…」
・COCRANE should be Cochrane. Make sure it is not a typo.
**Response:** Revised and Corrected
3. Materials and methods, Descriptions of the interested outcomes.
・What does the word “RBGL” mean? Please spell it in full when it first appears.

**Response:** Revised and spell it fully as Random blood glucose level (RBGL)

4. Results Prevalence of diabetic emergencies]
「Studies that carried out in China and Ethiopia has documented a range of prevalence rates or DKA episodes, from 3.8% to 73.4%, respectively (61, 74).」
4-1
・has→have……………..**Corrected**
Studies that carried out in China and Ethiopia have documented a range of prevalence rates or DKA episodes, from 3.8% to 73.4%, respectively (61, 74).
4-2
・“The prevalence of diabetic ketoacidosis (DKA) in the study by Ahmed MM et al., referenced as ref 77, was 83%.I think the ratio will be 3.8% to 83%.

**Response:** Thank you, its Revised based on your suggestion

5. Table 1
5-1
・”Sever Hypoglycemia” should be “Severe Hypoglycemia”.

**Response:** Corrected
5-2
・Cross sectional is described in multiple ways, so please unify.

**Response:** Revised and unified
5-3
The items I noticed are only a small part of the critical errors. The author should thoroughly review and correct the entire content.
・The studies included in this table have such varying definitions that they cannot be compared. Particularly, prevalence should be reconsidered.
〇Prevalence and Incidence
For example,
・The ref 59 Edo study, focuses on prevalence(58%).
・The ref.69 Abate study, focuses on incidence(0.9%).
・The ref.61 Negera study likely focuses on prevalence（73.4%).
・The ref.74 Li J study, on the other hand, deals with incidence(3.8%).
〇Different definition of Prevalence
・The ref.67 Gebre B study explores the prevalence of diabetes complications among patients with diabetes mellitus.The 61 Negera study specifically looks at the prevalence of diabetes complications among patients with DKA, HHS, and hypoglycemia.
・The ref.20 SarfoKantanka study focuses on the prevalence of diabetes complications among patients with DKA, HHS, hyperglycemia and hypoglycemia.
・However, the ref.77 Ahmed study focuses on the prevalence of diabetes complications among patients with DKA and HHS.
Therefore, these studies are not directly comparable due to their different approaches and definition. If the overall number is defined as different, it is natural that it is not possible to compare the diseases contained in it by proportion.

**Response:** *Thank you for highlighting this important concern. In response, we have added two columns in the table that describe the type of data of the included studies and definition of the diabetic emergencies in each study. We ensured that almost all of the studies included in the review used similar definitions of the diabetic emergencies (DKA, HHS and SH,) in line with the International Classification of Diseases (ICD) codes. This approach helped to standardize the definitions and improve comparability. Additionally, I have revised the manuscript to report prevalence data and incidence data separately, as you suggested. This separation will allow for clearer interpretation and minimize the potential for confusion when comparing studies with different methodological approaches*.

6. Discussion, Prevalence of diabetic emergencies
「The review encompassed 26 studies from Africa (n=14), Asia (n=5),and Mexico (n=1), revealing variations in DME prevalence both within and between countries in　the developing world, underscoring the considerable scope of the issue.」
・Wasn’t the number of the studies from Africa 20?

**Response**: yes! thank you, studies form Africa =20, it was a typo and corrected

**“That's all for my comments. Thank you.’’**

**Response to Reviewer #2**

Reviewer #2: The study is a systematic review of diabetic emergencies in developing countries. In total around 40 studies were included, the majority from Africa. Assessments of prevalence of emergencies and costs are presented, and a large variability is noted on study outcomes between the studies. The study addresses an important topic, but should be revised regarding language, structure and interpretation.
**Response:** *Thank you for your constructive comment we revised the Manuscript to address the objectives as much as possible.*
**Major:**
1. The study uses an unspecific language with frequent bold statements without support from real numbers eg. ” significant”, ”high incidence”, ”increased”, ”hazardous implications”, ”inadequate”. All such statements should be revised with quantified evidence from the references used in these sentences. A good example where this is already done is in the first paragraph of the introduction ”lower overall disease burden by 5-10%”.

Response: *Thank you for pointing out the need for greater specificity in language and the importance of supporting statements with quantified evidence. we have carefully reviewed the manuscript and revised instances where unspecific terms like "significant," "high incidence," "increased," and "inadequate" were used without numerical data or proper context. Wherever possible, we have incorporated quantifiable evidence from the references cited to substantiate these statements.*

2. With respect to the research question of the study, it may not be fair to include studies from developing countries across the globe, as there may be substantial differences between different regions. A sub-group analysis only including countries in a specific region could resolve this problem. This should at least be done examining outcomes only examining studies from Africa, where most studies were performed. Potential regional differences should also be further discussed in the study.

**Response:** *Thank you for your valuable suggestion regarding subgroup analysis by region. While we appreciate your concern about the potential substantial differences between regions and the implications this may have for the research, the included studies in this systematic review are not evenly distributed across regions, with 20 studies from Africa, 5 from Asia, and 1 from America. Given this imbalance, conducting a formal subgroup analysis would not provide meaningful or comparable insights due to the disproportionate representation of studies. To address this concern, we have already provided a qualitative description of the included studies in Table 1 and thoroughly discussed regional differences in the discussion section. Additionally, we have visually presented the regional distribution of studies by continent in Figure 2 to highlight this disparity.*

*We believe these approaches sufficiently address potential regional variations without performing a formal subgroup analysis, which may lead to misleading conclusions due to the limited number of studies from non-African regions.*

3. The current conclusion in the abstract is not drawn from the results in the study and must be revised (the conclusion of the manuscript is fair). Please consider who is the intended reader of this manuscript, descision makers? The large variability between studies prevents any strategic conclusion on policymaking. Which new studies should be conducted, and where? What interventions could reduce emergencies, and how should these interventions be tested? What does the estimated costs mean for the current treatment strategies, would it be cheaper to treat diabetes more effectively? These aspecst are currently not discussed sufficiently.

**Response:** *Thank you for your valuable feedback regarding the conclusion in the abstract. I agree that the current conclusion does not fully reflect the findings from the results of the study, and I will revise it to ensure it aligns more closely with the data presented. Additionally, I appreciate your suggestion to focus on the intended readers, particularly decision-makers, and the implications for policymaking.*

**Minor:**
1. There are some places where data is unclear. Eg the summary of the 26 studies is detailed but only adds up to 20 studies. All numbers must be doubble checked.

**Response**: Thank you! we checked and Revised
2. The cost of emergencies is estimated around 200 dollars. This is a quite normal anual medication cost for any patient in a western country, and is from this perspective not considered high. Therefore, some context must be provided for this number to make any sense. What is the annual income in these countries, what *proportion* of healthcare costs does this constitute?

**Response:** *Thank you for highlighting the need to contextualize the estimated cost of diabetic emergencies. we agreed that without proper context, the figure of $200 may not convey the full extent of the financial burden in developing countries. To address this, we already revised the manuscript to provide additional context regarding the economic impact of this cost. Specifically, we included data on the average annual income in the countries represented in the review, as well as the proportion of healthcare costs this figure represents for individuals and households in these settings.*
3. Hard numbers on the diabetes related mortalities in developing countries could further support the importance of this study.

**Response:** *Thank you for your insightful comment regarding the inclusion of diabetes-related mortality data. As the focus of my systematic review is specifically on diabetic emergencies, we prioritized including supportive data that highlights the severity and mortality impact of diabetic emergencies specifically Diabetic Ketoacidosis (DKA), Hyperosmolar Hyperglycemic State (HHS), and severe hypoglycemia in developing countries. These emergencies represent acute, life-threatening complications of diabetes that disproportionately contribute to mortality rates, particularly in resource-limited settings.* *By focusing on these specific outcomes, our aim is to provide a clearer understanding of the immediate risks and economic impacts posed by diabetic emergencies. Including additional context on diabetes-related mortality, as suggested, would have shifted the emphasis of the review from the targeted scope of diabetic emergencies to the broader burden of diabetes itself.*

4. Information regarding the current state of diabetes treatment in these regions is not discussed. CGM is mentioned, but is the measuring of glucose in patients available at all for these individuals? What proportion have access to diabetes medications, and what proportion can measure glucose or ketones themselves in any way?

**Response:** *Thank you for pointing out the importance of discussing the current state of diabetes treatment in the regions included in the review. While the primary focus of the study was on diabetic emergencies, I acknowledge that providing context about access to diabetes care and monitoring tools is crucial. Thus, I have revised the discussion section to include some details and highlighting the structural challenges that exacerbate the occurrence of diabetic emergencies in these regions.*

***Thank you all!***
