# Supplementary material for: Magnitude, risk factors and economic impacts of diabetic emergencies in developing countries: A systematic review
Source: PLoS One. 2025 Feb 4;20(2):e0317653. doi: 10.1371/journal.pone.0317653 (PMC11793792; doi:10.1371/journal.pone.0317653)
Supplement: Attachment — Submitted filename: 1. Response to Reviewers(MinorR).docx [file pone.0317653.s006.docx]

**Response to Reviewers for Minor Revision**

Dear Reviewers and Editor

*I hope this message finds you well. I would like to extend my sincere gratitude for your valuable feedback and suggestions on our manuscript. I appreciate the time and effort you have taken to review our work and provide constructive insights. We are currently addressing the comments and will ensure all necessary revisions are made to improve the quality of the manuscript. Please do not hesitate to let us know if there are any additional aspects you would like us to consider.*

*Thank you once again for your support and guidance.*

Journal Requirements:

Please review your reference list to ensure that it is complete and correct. If you have cited papers that have been retracted, please include the rationale for doing so in the manuscript text, or remove these references and replace them with relevant current references. Any changes to the reference list should be mentioned in the rebuttal letter that accompanies your revised manuscript. If you need to cite a retracted article, indicate the article’s retracted status in the References list and also include a citation and full reference for the retraction notice.

**Response:** *Thank you for your thorough review and valuable feedback on our manuscript. In response to your request, we have carefully reviewed the reference list to ensure that it is complete, accurate, and adheres to the journal's guidelines. After careful examination, we confirm that the reference list does not include any retracted articles. All cited references are current and relevant to the manuscript's content. we hope this addresses your concern effectively.*

**Response to Additional Editor Comments**

Dear authors, please make the following changes to the manuscript, particularly those enlisted by referee 2.

Here are some of my specific considerations: 
1. Across all text, consider using the term “adults living with diabetes” instead of "adults with DM" for clarity and consistency.

**Response:** Corrected

2. In the abstract, correct the typo in the phrase "Sever hypoglycemia" to "Severe hypoglycemia."

**Response:** *Its corrected including in other parts*
3. In the introduction section, in the line “non-communicable diseases (NCDs) are expected to increase and surpass infectious diseases by 2030, according to predictions," specify the source of predictions—are they from the NCD risk collaboration or the GBD studies?

**Response:** *Source specified (i.e. GDB studies) and the sentences has rephrased*.
4. Conduct a thorough check for typos and extra spaces throughout the manuscript. Examples include “Diabetes Mullites” and “irrelevant conte," as well as "sever hypoglycemia."

**Response:** *Thank you, I checked and corrected*
5. Please revise and ensure that the citations adhere to PLOS ONE guidelines, as some citations do not include the journal name or the year of publication.

**Response:** *References are revised and made to adhere to PLOS ONE guidelines*

**Response to Reviewer #1**

【Minor comments】
1)(Introduction)
・The global incidence of diabetes is rising rapidly, posing a significant public health challenge, particularly in developing countries, which account for approximately 80% of the 537 million people living with diabetes worldwide as of 2021.
〇 Please add the reference of the facts.

**Response**: References added

2)(Introduction)
〇 The sentence 「it characterized by abnormally high or low blood sugar levels, are significant contributors to morbidity, mortality, and increased healthcare costs among adults with diabetes. For instance, diabetic ketoacidosis (DKA) and hyperosmolar hyperglycemic state (HHS) collectively account for up to 30% of diabetes-related hospital admissions in developing countries, with mortality rates ranging from 5% to 20% depending on the region and healthcare access. may be 「it is characterized by abnormally high or low blood sugar levels, are significant contributors to morbidity, mortality, and increased healthcare costs among adults with diabetes. For instance, diabetic ketoacidosis (DKA) and hyperosmolar hyperglycemic state (HHS) collectively account for up to 30% of diabetes-related hospital admissions in developing countries, with mortality rates ranging from 5% to 20% depending on the region and healthcare access.

**Response**: Accepted and Rewrote

3)(Introduction)
〇The sentence「Even though diabetic emergencies are becoming more common in poor nations, there is a conspicuous Visible scarcity of paucity of thorough in-depth studies in this area, and the research that is currently available lacks statistical clarity and specificity. may be [Even though diabetic emergencies are becoming more common in poor nations, there is a conspicuous visible scarcity of paucity of thorough in-depth studies in this area, and the research that is currently available lacks statistical clarity and specificity.].

**Response**: Accepted and Rewrote

4)(Results, Table 1)
The authors are calculating the prevalence, but the denominators differ.
・The ref.67 Gebre B study explores the prevalence of diabetes complications “among patients with diabetes mellitus.” The denominators are patients with diabetes mellitus.
However, the 61 Negera study specifically looks at the prevalence of diabetes complications “among patients with DKA, HHS, and hypoglycemia”. The denominators are not the patients with diabetes, but rather the patients with DKA, HHS, and hypoglycemia.
・The ref.20 SarfoKantanka study focuses on the prevalence of diabetes complications “among patients with DKA, HHS, hyperglycemia and hypoglycemia”. The denominators are not the patients with diabetes, but rather the patients with DKA, HHS, hyperglycemia, and hypoglycemia.

→〇 If the groups of diseases included as subjects differ in each study, then each prevalence cannot be compared.

**Response:** *Thank you for pointing out the issue regarding the comparability of prevalence across studies that involve different groups of diseases. I acknowledge that comparing prevalence rates directly between studies with differing denominators (e.g., patients with diabetes mellitus vs. those with DKA, HHS, or hypoglycemia) can lead to misleading conclusions. To address this concern, I have revised the manuscript as follows:*

1. *We clearly stated in the text (in the result section of the study) that the groups of diseases and the denominators differ among the studies, making direct comparisons inappropriate.*
2. *We adjusted the discussion section to emphasize that the prevalence findings should be interpreted within the context of each study’s population and methodology.*
3. *We didn’t use any implications in the manuscript that might suggest direct comparisons between these prevalence rates. I hope these revisions adequately address your concern and ensures that the interpretations are valid, and the readers are guided appropriately.*

5)(Results, Table 4)

〇 Please fill in the blanks in the Total score 'Yes (Y).'

**Response:** *total score yes(Y) filled=6*

6) Overall

〇 Please review the document overall for minor English mistakes.
Example: Descriptions of the Interested Outcomes

・Severe Hypoglycemia → Severe Hypoglycemia.

**Response:** Document reviewed for minor language mistakes.
